# Supplementary figures and images for: A phage-encoded counter-defense inhibits an NAD-degrading anti-phage defense system
Source: PLoS Genet. 2025 Apr 2;21(4):e1011551. doi: 10.1371/journal.pgen.1011551 (PMC11984713; doi:10.1371/journal.pgen.1011551)

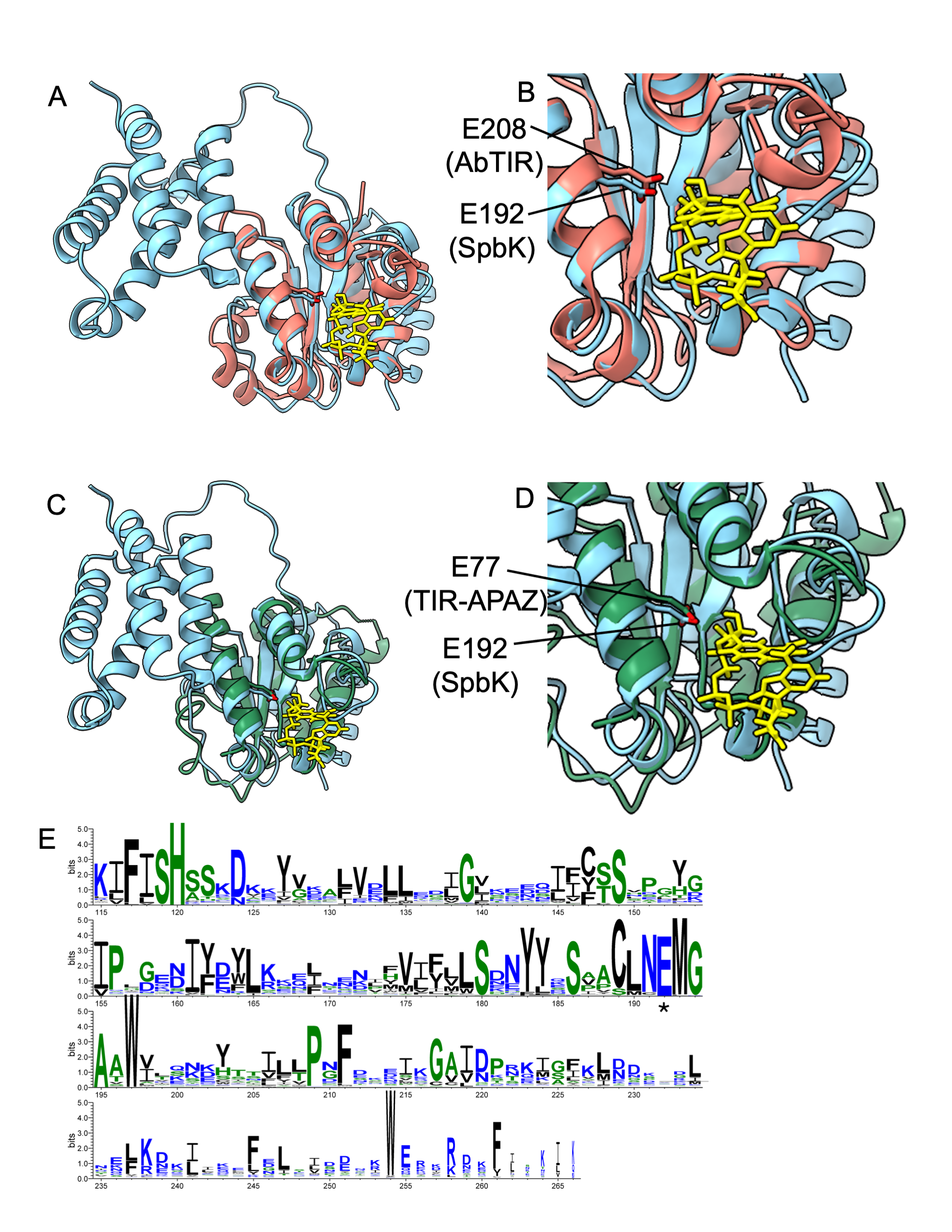

Supplement: S1 Fig — A, B) An AlphaFold3 model of an SpbK (blue) monomer overlayed with AbTIR (pink; PDB: 7UXU). The NAD molecule is colored yellow. B) An enlarged view of the NAD binding pocket from A. C, D) An AlphaFold3 model of an SpbK (blue) monomer overlayed with the TIR domain from TIR-APAZ (green; PDB: 8I87). The NAD molecule is colored yellow. D) An enlarged view of the NAD binding pocket from C. E) Sequence logo of the 500 highest scoring proteins from PSI-BLAST using SpbK as the query. The TIR domain of SpbK (amino acids 115–266) are shown. The highly conserved glutamate in the TIR domain NADases, E192 in SpbK, is marked with an asterisk (*). (TIF) [file pgen.1011551.s001.tif]

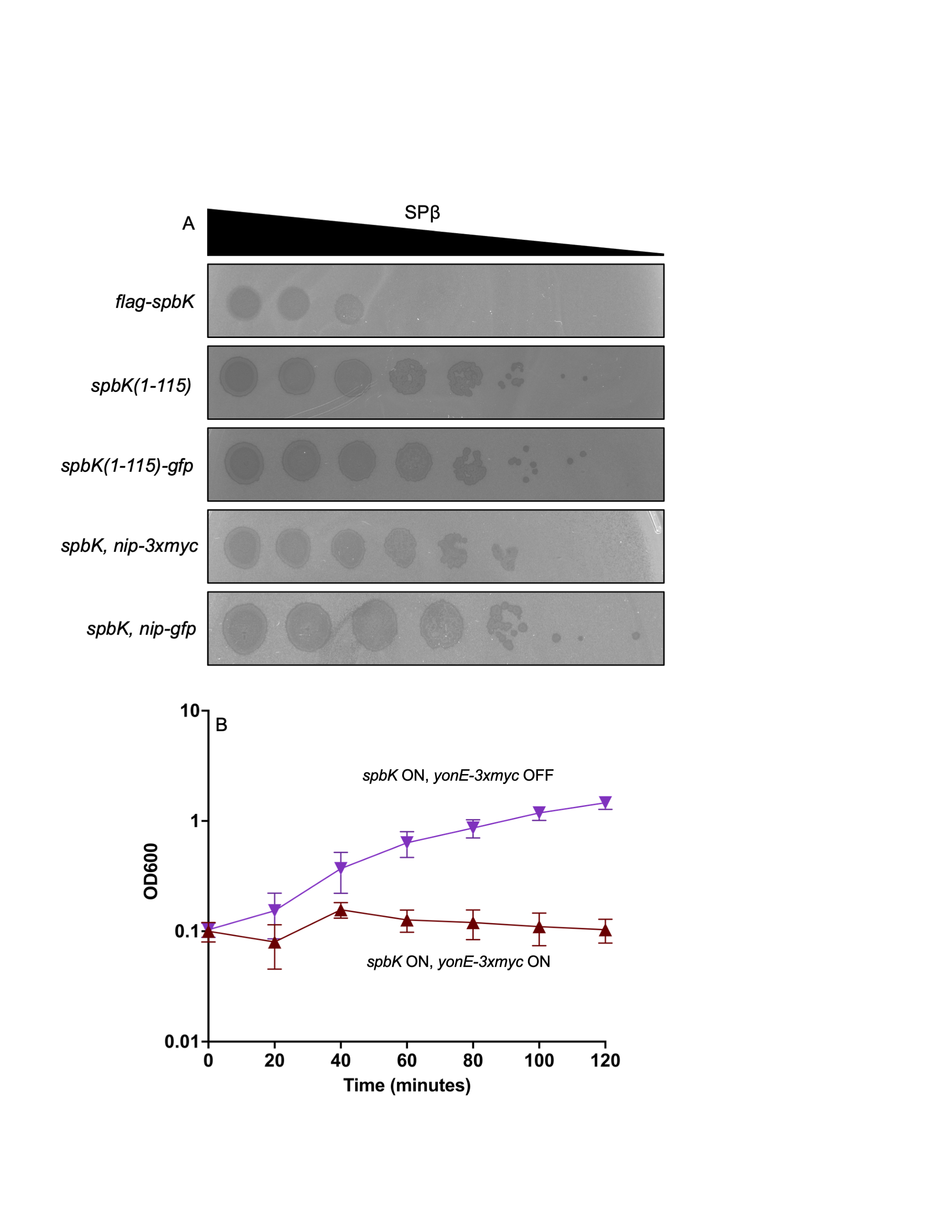

Supplement: S2 Fig — A) Fusion proteins Nip-Myc (top row) and Nip-GFP (second row) were co-expressed with wild type SpbK. FLAG-SpbK (third row), SpbK(1–115) (fourth row), and SpbK(1–115)-GFP (fifth row) were expressed alone. Ten-fold dilutions of SPβ were spotted and functionality was assessed by observing presence or absence of phage plaques. Large zones of clearing are indicative of a confluence of phage plaques and cell lysis. Small zones of clearing are indicative of individual or small clusters of phage plaques. B) YonE-Myc causes growth arrest when co-expressed with wild type SpbK. Strains co-expressing spbK and yonE-3xmyc were grown in LB medium. Turbidity of the culture was measured by OD600 and followed over time when yonE-3xmyc was induced with 1mM IPTG (maroon triangles) or left uninduced (purple inverted triangles). Measurements at T=0 were taken immediately before addition of IPTG. Error bars represent standard deviation. Data shown are from three biological replicates. Error bars represent standard deviation and are not always depicted due to the size of the data point. (TIF) [file pgen.1011551.s002.tif]

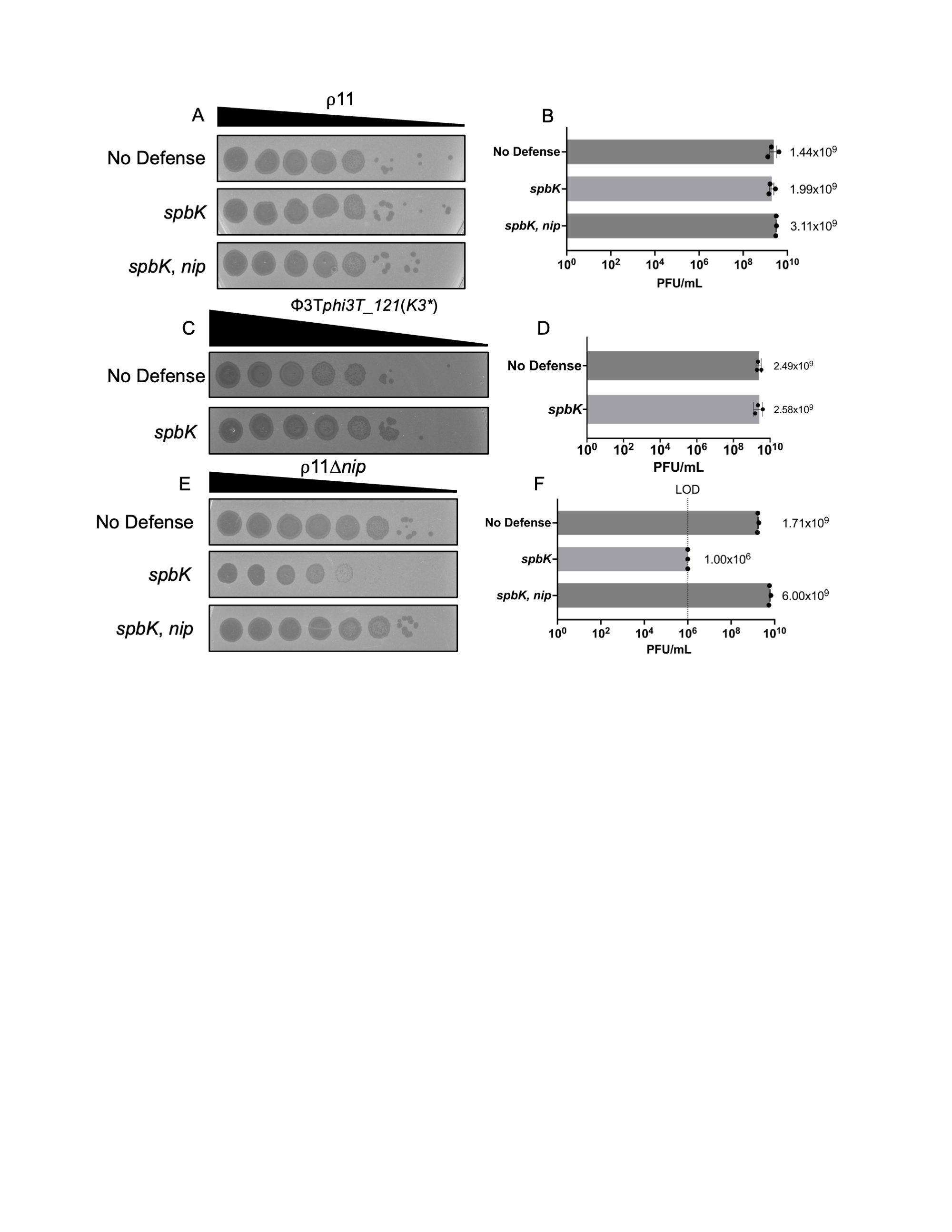

Supplement: S3 Fig — A, B) ρ11 evades SpbK-mediated anti-phage defense. A) Ten-fold dilutions of ρ11 were spotted onto isogenic strains without phage defense (CU1050, top), expressing spbK (CMJ534, middle), or co-expressing spbK and nip (CLL356; bottom). Large zones of clearing indicate cell lysis while small zones of clearing are indicative of productive phage infection. ρ11 was plated with isogenic strains expressing no anti-phage defense (CU1050, top bar), spbK (CMJ534, middle bar), or spbK and nip together (CLL356, bottom bar). Error bars represent standard deviation and are not always depicted due to the size of the data point. C, D) Φ3Tphi3T_121(K3STOP) still has counter-defense against SpbK. C) Ten-fold dilutions of Φ3Tphi3T_121(K3STOP) were spotted onto isogenic strains lacking anti-phage defense (CU1050, top row) or expressing spbK (CMJ534, bottom row). Large zones of clearing indicate cell lysis while small zones of clearing are indicative of productive phage infection. D) Strains lacking anti-phage defense (black bar) or expressing spbK (pink bar) were infected with Φ3Tphi3T_121(K3STOP). Error bars represent standard deviation and are not always visible. E, F) nip is required for ρ11 evasion of SpbK-mediated anti-phage defense. C) Ten-fold dilutions of ρ11∆nip were spotted onto isogenic strains without phage defense (CU1050, top), expressing spbK (CMJ534, middle), or co-expressing spbK and nip (CLL356; bottom). Large zones of clearing indicate cell lysis while small zones of clearing are indicative of productive phage infection. ρ11 was plated with isogenic strains expressing no anti-phage defense (CU1050, top bar), spbK (CMJ534, middle bar), or spbK and nip together (CLL356, bottom bar). Error bars represent standard deviation and are not always depicted due to the size of the data point. The limit of detection in these experiments was ~106 PFU/ml. (TIF) [file pgen.1011551.s003.tif]

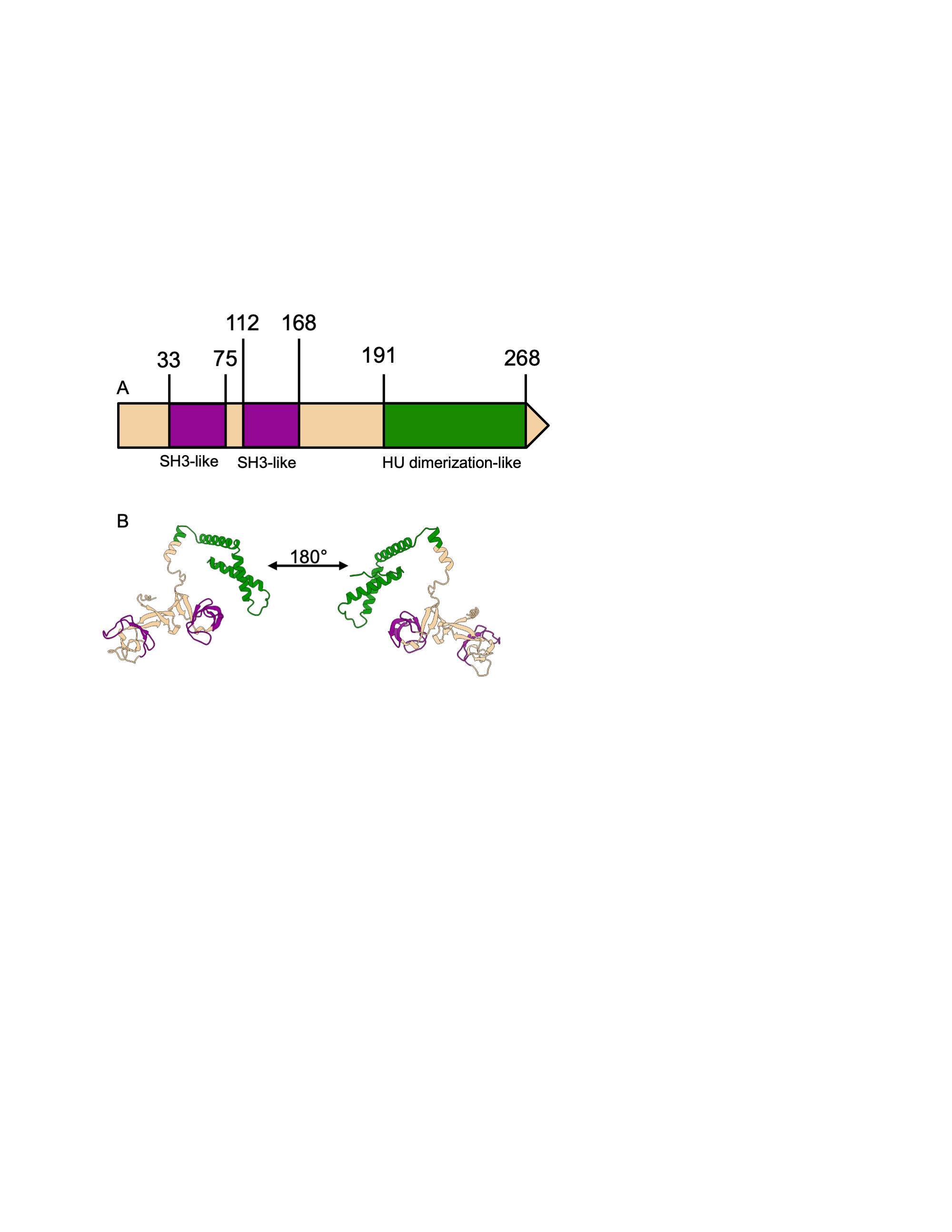

Supplement: S4 Fig — A) Predicted domain architecture of Nip. Amino acid positions are indicated at the top and regions of similarity to other proteins indicated at the bottom. Nip is 279 amino acids and predicted to have two SH3-like domains (purple; aa 33–75 and 112–168) and an HU dimerization-like domain (green; aa 191–268). B) AlphaFold3 model of a monomer of Nip. The SH3-like domains are colored purple and the HU dimerization-like domain is colored green. The pTM score is 0.57 and the mean pLDDT score is 75.62, indicating low-moderate confidence in the model. (TIF) [file pgen.1011551.s004.tif]

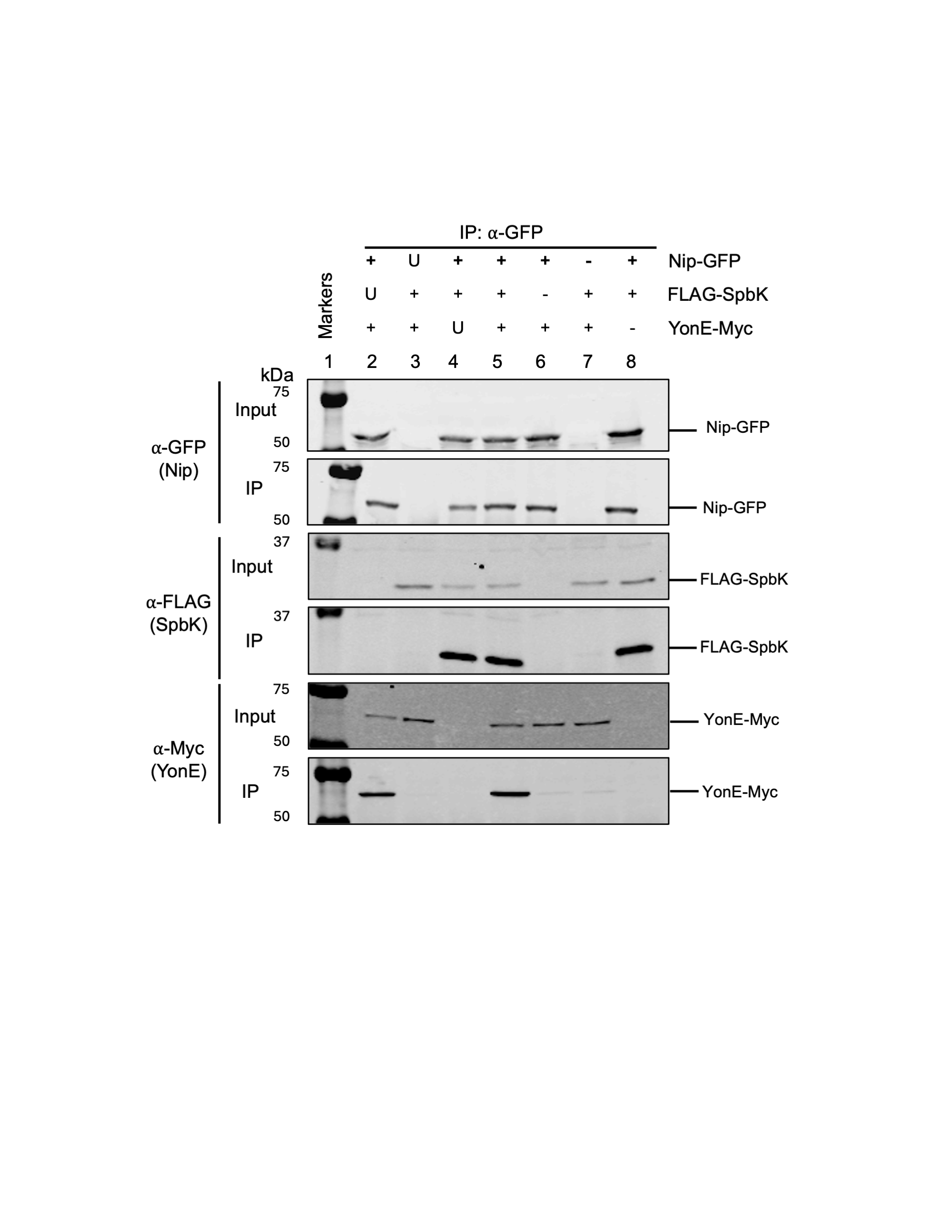

Supplement: S5 Fig — Data for the immunoprecipitation experiments are the same as in Fig 7 and are shown here for comparison. Input samples are shown in the panel above each of the corresponding immunoprecipitations (IP). Indicated proteins were expressed in cells without ICEBs1 or SPβ. First and second rows: Western blots probed with ⍺-GFP polyclonal antibodies on input samples and immunoprecipitates, respectively. Third and fourth rows: Western blot probed with ⍺-FLAG monoclonal antibodies on input samples and immunoprecipitates, respectively. Fifth and sixth rows: Western blot probed with ⍺-c-Myc monoclonal antibodies on input samples and immunoprecipitates, respectively. Lane 1 contains molecular weight markers from the Odyssey One-Color Protein Molecular Weight Marker Ladder (LI-COR). Lysates and IPs were from strains: CLL633 (lane 2), CLL642 (lane 3), and CLL704 (lane 4), CLL498 (lane 5), CLL497 (lane 6), CLL373 (lane 7), and CLL382 (lane 8). Data shown are representative of three biological replicates. U, untagged protein expressed; +, tagged protein expressed; -, protein not expressed. (TIF) [file pgen.1011551.s005.tif]
